# Desmoglein‐2 expression is an independent predictor of poor prognosis patients with multiple myeloma

**DOI:** 10.1002/1878-0261.13055

**Published:** 2021-07-24

**Authors:** Lisa M. Ebert, Kate Vandyke, M. Zahied Johan, Mark DeNichilo, Lih Y. Tan, Kay K. Myo Min, Benjamin M. Weimann, Brenton W. Ebert, Stuart M. Pitson, Andrew C. W. Zannettino, Craig T. Wallington‐Beddoe, Claudine S. Bonder

**Affiliations:** ^1^ Centre for Cancer Biology SA Pathology and University of South Australia Adelaide SA Australia; ^2^ Adelaide Medical School Faculty of Health and Medical Sciences The University of Adelaide Adelaide SA Australia; ^3^ Myeloma Research Laboratory Precision Medicine Theme South Australian Health and Medical Research Institute Adelaide SA Australia; ^4^ College of Medicine and Public Health Flinders University Bedford Park SA Australia; ^5^ Flinders Medical Centre Bedford Park SA Australia

**Keywords:** adhesion, bone marrow, desmoglein‐2, multiple myeloma, plasma cells, prognostic

## Abstract

Multiple myeloma (MM) is the second most common haematological malignancy and is an incurable disease of neoplastic plasma cells (PC). Newly diagnosed MM patients currently undergo lengthy genetic testing to match chromosomal mutations with the most potent drug/s to decelerate disease progression. With only 17% of MM patients surviving 10‐years postdiagnosis, faster detection and earlier intervention would unequivocally improve outcomes. Here, we show that the cell surface protein desmoglein‐2 (DSG2) is overexpressed in ~ 20% of bone marrow biopsies from newly diagnosed MM patients. Importantly, *DSG2* expression was strongly predictive of poor clinical outcome, with patients expressing *DSG2* above the 70^th^ percentile exhibiting an almost 3‐fold increased risk of death. As a prognostic factor, DSG2 is independent of genetic subtype as well as the routinely measured biomarkers of MM activity (e.g. paraprotein). Functional studies revealed a nonredundant role for DSG2 in adhesion of MM PC to endothelial cells. Together, our studies suggest DSG2 to be a potential cell surface biomarker that can be readily detected by flow cytometry to rapidly predict disease trajectory at the time of diagnosis.

## Graphical abstract

This study suggests that desmoglein‐2 (DSG2) is a novel cell surface adhesion molecule expressed by neoplastic plasma cells of patients with multiple myeloma. It presents a clear link to poor prognosis of myeloma patients across all cytogenetic subtypes. DSG2 is a readily measurable and clinically useful prognostic biomarker that may prove to be a novel target to combat myeloma progression and improve patient outcomes.

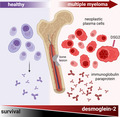

**Abbreviations**
BM: bone marrow
BMEC: BM endothelial cell
CCND1: CD1, cyclin D1
CCND3: CD2, cyclin D3
DSC: desmocollin
DSG2: desmoglein‐2
FFPE: formalin‐fixed paraffin‐embedded
FMO: fluorescence minus one
HR: hazard ratio
HY: hyperdiploid
IFM: immunofluorescence microscopy
IHC: immunohistochemistry
LB: low bone disease
MF: MAF/MAFB
MM: multiple myeloma
MMP: matrix metallopeptidase
MMSET: MS, multiple myeloma SET domain
ORR: overall response rates
OS: overall survival
PB: peripheral blood
PC: plasma cells
PFS: progression‐free survival
PR: proliferative
siRNA: small interfering ribonucleic acid
TrHBMEC: trabecular human BM endothelial cells

## 1 Introduction

Multiple myeloma (MM) is an incurable malignancy of neoplastic antibody‐secreting plasma cells (PC), with a median age at diagnosis of 69 years and a median overall survival of 6–7 years [1]. With an age‐adjusted incidence of six per 100 000 per year in the USA and Europe, it is the second most common haematological cancer [1]. The past two decades have seen the introduction of novel agents that have dramatically improved overall response rates (ORR), progression‐free survival (PFS) and overall survival (OS) for MM patients; however, disease relapse generally occurs, and the disease is currently incurable.

The ability to stratify MM patients, based on the biology of their disease, is critical in guiding appropriate therapy and clinical monitoring of an individual’s risk of disease progression [2, 3]. For example, the t(4;14) chromosomal translocation occurs in approximately 15% of MM patients and is associated with intermediate to poor prognosis compared to patients without this translocation [4]. Specifically, t(4;14)‐positive MM is characterized by rapid disease progression and disease relapse, and increased tumour dissemination, reflected by an increase in the number circulating PC in the peripheral circulation [5, 6]. Staging systems, such as the revised international staging system (R‐ISS), have been developed in order to improve treatment decisions. However, their utility in the era of an ever‐increasing repertoire of novel agents to treat MM requires continual refinement to maintain prognostic validity. New appropriate biomarkers to achieve this goal are thus needed [2].

Desmoglein‐2 (DSG2) is a surface‐expressed adhesion molecule belonging to the cadherin family primarily known for its function in the formation of cell–cell adhesion multiprotein complexes known as desmosomes, which are found in simple and stratified epithelia and myocardium [7, 8]. In humans, four desmoglein isoforms (DSG1‐4) have been identified which, together with members of the closely related desmocollin family (DSC1‐3), undergo calcium‐dependent homotypic and heterotypic interactions to generate the adhesive interface of desmosomes between adjacent cells. Collectively, these molecules are known as desmosomal cadherins.

Amongst the desmosomal cadherins, DSG2 is gaining recognition for its ability to exist outside of desmosomes and to regulate additional biological processes [9, 10, 11]. For example, an intracellular fragment of DSG2 can regulate caspase‐3 cleavage and apoptosis in intestinal epithelial cells [9], while overexpression of DSG2 in suprabasal keratinocytes has been shown to induce hyperproliferation, resistance to anoikis and enhanced carcinogenesis [10]. Furthermore, studies by our laboratory and others have demonstrated a role for DSG2 in regulating multiple aspects of endothelial cell biology, including barrier function and angiogenic activity [12, 13], and in promoting vasculogenic mimicry activity of human melanoma cells [14]. These findings suggest a prominent role for DSG2 in regulating cell adhesion and vascular function.

Intriguingly, DSG2 can also be expressed within the haematopoietic compartment, where expression is restricted to stem and progenitor populations. More specifically, expression is detectable on human haematopoietic stem/progenitor cells within adult blood, umbilical cord blood and normal bone marrow (BM), but is rapidly lost during differentiation to mature leukocyte populations [12]. This highlights the potential novel biological roles for DSG2, particularly considering that haematopoietic cells lack desmosomes.

Here, we demonstrate that DSG2 is strongly up‐regulated on the surface of neoplastic PC in a distinct subset of MM patients. The expression of DSG2 is associated with a striking reduction in progression‐free and overall survival of MM patients, thus revealing DSG2 as a novel biomarker of poor prognosis with potential clinical utility. In addition, we show that DSG2 directly contributes to adhesive interactions between MM PC and BM endothelial cells, which may support the dissemination of MM PC to new sites within the BM.

## 2 Materials and methods

### 2.1 Cell lines and culture

Human MM cell lines LP‐1, KMS‐11, RPMI8226 and U266 were obtained from the American Type Culture Collection (ATCC, VA, USA); OPM2, MM.1S, MM.1R and NCI‐H929 were kindly provided by Prof. Andrew Spencer (Monash University, Vic, Australia); KMS‐18 were kindly provided by Prof. Junia Melo (SA Pathology, SA, Australia). MM cell lines were maintained in culture in a semi‐adherent state in RPMI1640 media (Gibco, Thermo Fisher Scientific, Waltham, MA, USA) supplemented with 10% FBS (HyClone, Logan, UT, USA) and 2 mm GlutaMax (Gibco). The immortalized human BM endothelial cell line TrHBMEC [15] was a kind gift from B Weksler (Cornell University Medical College, NY, USA) and was cultured in HUVE medium as described [15]. All cultures were periodically confirmed negative for mycoplasma using MycoAlert (Lonza, Basel, Switzerland).

### 2.2 DSG2 knockdown in human MM cell lines and cell proliferation analysis

Lentiviral vectors (pGIPZ) expressing DSG2‐shRNA and nonsilencing control‐shRNA were obtained from Open Biosystem (Dharmacon, Lafayette, CO, USA) and cloned into the pGIPZ expression plasmid: 5′‐AGG GTTTTAGTTGTCCTGA‐3′ (DSG2‐shRNA_A); 5′‐CCAGTGTTCTACCTAAATA‐3′ (DSG2‐shRNA_B); and 5′‐ ATCTCGCTTGGGCGAGAGTAAG‐3′ (nonsilencing shRNA). Replication incompetent lentiviral particles were generated by transiently co‐transfecting HEK293T cells with ViraPower Lentiviral Support Kit (Invitrogen, Carlsbad, CA, USA) and pGIPZ‐shRNA vectors using Lipofectamine 2000 (Invitrogen). Lentiviral supernatant was harvested 72 h post‐transfection and used to transduce 1 × 10^5^ KMS‐11 cells that were seeded in a 6‐well plate, in the presence of 4 µg·mL^−1^ polybrene. Puromycin (1 µg·mL^−1^, Gibco) was continually added to maintain culture of cells with the transduced vectors and DSG2 expression was routinely checked using flow cytometry and western blot. The metabolic activity of cells was compared following 72 h of cell culture and assessed over 60 min at 37 °C using the alamarBlue fluorescent dye assay (Invitrogen) with fluorescence intensity measured (530‐nm ex and 595‐nm em) using a FLUOstar Optima plate reader (BMG Labtech, Mornington, Vic, Australia).

Transient silencing of DSG2 expression on the surface of RPMI8226 cells was achieved by treating 2.5 × 10^5^ cells for 72 h with 10 nm of DSG2‐targeting 27mer siRNA duplexes (SR301282, OriGene, Rockville, MD, USA) delivered using the Lipofectamine RNAiMAX transfection reagent (Invitrogen). As a control, cells were also treated with 10 nm of the Universal nonsilencing siRNA duplex (OriGene) for 72 h. DSG2 expression was routinely checked using flow cytometry and western blot. Cell numbers were compared over 72 h following knockdown and assessed using alamarBlue as detailed above.

### 2.3 Patient samples

We used cryopreserved peripheral blood (PB), posterior superior iliac spine BM aspirates and trephine biopsies from newly diagnosed MM patients, as defined by standard diagnostic criteria [16] (median age: 64 years [range 41–81]; male:female, 8 : 9). Ethical approval for this cohort was obtained from the Royal Adelaide Hospital Human Research Ethics Committee (approval numbers 030206, 131132 and 110304), and all participants provided written informed consent. We also used prospectively collected PB and posterior superior iliac spine BM aspirate from 54 newly diagnosed MM patients at Flinders Medical Centre, Australia, with median age: 67 years [range 42–85]; male : female, 36 : 18. Ethical approval for this cohort was obtained from Southern Adelaide Local Health Network Human Research Ethics Committee (approval HREC/18/SAC/301) and all patients provided written informed consent. The study methodologies conformed to the standards set by the Declaration of Helsinki.

### 2.4 Flow cytometry

DSG2 was assessed by flow cytometry on viable CD38^++^CD138^+^CD45^lo^CD19^‐^ MM PC, as previously described [17]. Patient BM and PB mononuclear cells were stained with anti‐DSG2 antibody (clone 6D8, Invitrogen) or no primary antibody [fluorescence minus one (FMO) control] followed by a PE‐goat anti‐mouse IgG secondary antibody (Southern Biotech, Birmingham, AL, USA) prior to staining with antibodies CD38‐PE‐Cy7 (HIT2; BioLegend, San Diego, CA, USA), CD138‐AlexaFluor‐647 (B‐B4; Serotec, Oxford, UK), CD45‐FITC (J.33; Beckman Coulter, Brea, CA, USA), CD19‐Brilliant Violet 421 (HIB19; BioLegend) and the viability dye hydroxystilbamidine (FluoroGold; Invitrogen). For the prospective analysis of newly diagnosed patients at Flinders Medical Centre, patient BM was stained with DSG2‐Alexa Fluor 488 (CSTEM28; Invitrogen) or no primary antibody [fluorescence minus one (FMO) control] together with antibodies CD38‐V450 (HB7; BD Biosciences, Franklin Lakes, NJ, USA), CD138‐PE (MI15; BD Biosciences), CD45‐PerCP‐Cy5.5 (HI30, BD Biosciences) and CD19‐PE‐Cy7 (SJ25C1; BD Biosciences). For analysis of DSG2 expression on MM cell lines, cells were incubated with either Alexa Fluor 488‐conjugated anti‐DSG2 monoclonal antibody (clone CSTEM28), unconjugated monoclonal antibodies to DSG2 (clone 6D8 (IgG1, Invitrogen) or clone 9F6 (IgG)). The anti‐human DSG2 clone 9F6 was newly developed at the Monash Antibody Technologies Facility (Melbourne, Vic, Australia) wherein Monash University Animal Ethics Committee approved 6‐week‐old Robertsonian mouse intraperitoneal injection with recombinant DSG2 protein (32 µg in Sigma Adjuvant System, Sigma‐Aldrich, St. Louis, MO, USA), with three boosts given every 2 weeks for 6 weeks. After the final boost, mice with serum reactivity were identified and given an intravenous prefusion boost. Five days later, primary splenocytes were isolated and fused with myeloma Sp2/0 cells. Cells were plated onto 96‐well plates to generate antibody‐producing hybridomas which were screened for high‐affinity‐specific DSG2 antigen‐positive lines using microarray (Arraviet Super Marathon, ArrayJet, Roslin, UK) and standard ELISA. Flow cytometry samples were analysed on a LSRFortessa, a FACS Canto II or an Accuri C6 flow cytometer (BD Biosciences) and data analysed using FlowJo v10.7.1 (BD Biosciences) or FCS Express 4 Flow Cytometry: Research Edition (De Novo Software, Glendale, CA, USA).

### 2.5 Immunohistochemistry and immunofluorescence microscopy

Formalin‐fixed, decalcified, paraffin‐embedded (FFPE) trephine biopsies from three newly diagnosed MM patients or a healthy control were sectioned, dewaxed and subjected to heat‐mediated antigen retrieval (20 min in a microwave) in pH 6.0/6.5 sodium citrate buffer. Immunohistochemistry (IHC) was performed using the ADVANCETM HRP polymer system kit (Dako, Glostrup, Denmark) according to the manufacturer’s recommendations wherein endogenous peroxidase block was used for 30 min at RT prior to 60 min with primary mAb against DSG2 (0.9 µg·mL^−1^ final concentration, clone #141409, MAB947 R&D Systems, Minneapolis, MN, USA), anti‐CD138 (clone MI15; Dako, 1 : 100 dilution from stock), anti‐CD31 (clone 89C2; Cell Signaling Technology, Danvers, MA, USA, 1 : 1200 dilution from stock) or an isotype‐matched (IgG1) control antibody (0.5 µg·mL^−1^, Abcam, Cambridge, UK), followed by reaction with DAB, counterstaining using Mayer’s haematoxylin and mounting in DPX. Images were captured via an inverted DP80 photographic microscope (Olympus, Tokyo, Japan).

For immunofluorescence microscopy (IFM), sections were blocked with 10% normal goat serum (Sigma‐Aldrich) made up in a CAS block buffer for 1 h (Life Technologies, Carlsbad, CA, USA) then incubated overnight at 4°C with primary mAb against DSG2 (1 µg·mL^−1^, clone 141409, R&D Systems) and an anti‐CD138 (clone 359103; R&D Systems, 1 : 50 dilution from stock), followed by relevant secondaries conjugated to fluorochromes Alexa‐488 or Alexa‐555 (1 : 500, Life Technologies) for 1 h. Detection was performed using an LSM700 laser scanning confocal microscope (Zeiss, Oberkochen, Germany) and counterstaining using DAPI (1 µg·mL^−1^, Sigma‐Aldrich) and mounted in Fluoro‐Gel, water‐based mounting medium (ProSciTech, Kirwan, QLD, Australia).

### 2.6 DSG2 ELISA

Myeloma patient blood was collected in CAT Serum Sep Clot Activator tubes (Greiner‐Bio One, Kremsmunster, Austria) and allowed to clot before centrifugation at 1200 ***g*** for 10 min. Serum was aliquoted and stored at −80 °C before use. Serum samples from 13 myeloma patients with a range of DSG2 expression levels on MM PC by flow cytometry were tested for soluble DSG2 (sDSG2) by ELISA (#ELH‐DSG2, RayBiotech, Norcross, GA, USA), along with serum from 5 healthy controls. ELISA was performed per manufacturer’s directions. Briefly, samples were diluted (1 : 1) in assay diluent before incubation at RT for 150 min, followed by sequential incubations with biotinylated secondary antibody, streptavidin solution and TMB one‐step substrate reagent for 60, 45 and 10‐15 min, respectively. Wells were washed four times between reagents with wash buffer. Stop reagent was added after the TMB reagent and plates were immediately read at 450 nm. All myeloma samples were run in triplicate, and standard curves were run in duplicate.

### 2.7 Adhesion assay

BMEC were seeded in 2.5 mL of HUVE media onto 35 × 10 mm culture dishes (Corning) until confluent. KMS‐11 cells (1 × 10^6^ ± DSG2‐targeting GFP‐tagged shRNAs) or RPMI8226 cells (1 × 10^6^ ± DSG2‐targeting siRNAs and labelled with the calcein AM viability dye (5 μg·mL^−1^; eBiosciences, San Diego, CA, USA)) were added onto the BMEC monolayer in 1 mL of HBSS for 15 min at 37 °C and 5% CO_2_, the HBSS was aspirated, and dishes rinsed 2 × 2 mL of HBSS to remove unbound cells prior to a final wash on an orbital mixer (Ratek, Boronia, Vic., Australia) at a speed setting of 5. Unbound cells were aspirated, and 1ml of fresh HBSS was added to the dishes. Fluorescent images were taken at 7–8 fields of views across the middle of the dish under a 10×/0.30 objective on an IX73 inverted fluorescent microscope (Olympus) using the cellSens Dimension software (Olympus). The number of bound fluorescent cells was quantified using image j (NIH, Bethesda, MD, USA).

### 2.8 Lysate preparation and western blotting

Washed KMS‐11 and RPMI8226 cells solubilized in RIPA lysis buffer containing protease (cOmplete™, Roche, Basel, Switzerland) and phosphatase inhibitors (PhosStop™, Roche) for 10 min on ice. Lysates were clarified and boiled in reducing SDS sample buffer for 5 min. Samples (50 μg per lane) were resolved in 4–12% Bis‐Tris polyacrylamide gels (Bio‐Rad, Hercules, CA, USA) and electrophoretically transferred to nitrocellulose filters (Pall Corporation, New York, NY, USA) prior to blocking (Odyssey Blocking Buffer, Li‐COR, Lincoln, NE, USA) and incubation with antibodies to DSG2 (Bethyl Laboratories, Montgomery, TX, USA), phospho‐p44/42 MAPK (ERK1/2, Thr202/Tyr204; CST), phospho‐AKT (Ser473; CST) or IƘBα (Cell Signaling Technology) at 1 : 1000 dilution in blocking buffer for 1 h. Washed filters were incubated for 45 min with either IRDye 800CW goat anti‐rabbit IgG (Li‐COR) or IRDye 680CW goat anti‐mouse at a 1 : 10 000 dilution in blocking buffer. Immunoreactivity was detected (Odyssey infrared imager, Li‐COR) and filters stripped and re‐probed with antibodies to GAPDH, p44/42 MAPK (Erk1/2) or AKT (Cell Signaling Technology). Band intensities were quantitated by densitometry (Odyssey infrared imager, Li‐COR).

### 2.9 Analysis of DNA microarray and RNAseq datasets

*DSG2* gene expression was assessed (as described [18]) in CD138‐selected human BM PC from patients with newly diagnosed MM, monoclonal gammopathy of undetermined significance (MGUS) and healthy controls in microarray datasets E‐GEOD‐16122 (normal, *n* = 5; MGUS, *n* = 11; MM, *n* = 133; PCL, *n* = 9) [19], E‐MTAB‐363 (normal, *n* = 5; MGUS, *n* = 5; MM, *n* = 155) [20] and RNAseq data from a panel of 65 human myeloma cell lines [21]. Dataset GSE4581 was used to assess the link between *DSG2* expression and overall survival, and to perform differential gene expression analyses [22]. Data were downloaded in R with the aid of the GEOquery library [23], log2 transformed and analysed in Bioconductor using limma library [24] to perform differential gene analysis and pHeatmap library. Individual samples were assigned to subsets (MS, CD1, CD2, LB, HY, MF or PR) according to labels provided by the data owner, as described previously [22]. The coMMpass study RNAseq datasets of MM patients treated outside of clinical trials were log2 transformed and used to assess links between *DSG2* expression in CD138‐selected human BM PC and progression‐free survival, overall survival and MM drug class used as frontline therapy (proteasome inhibitor or immunomodulatory agent). These data were generated as part of the Multiple Myeloma Research Foundation Personalized Medicine Initiatives (https://research.themmrf.org and www.themmrf.org).

### 2.10 Myeloma cell drug treatment

Myeloma cells (KMS‐11 and RPMI8226 ± DSG2‐targeting sh/siRNAs) were seeded into 96‐well plates at a density 6 × 10^4^ cells per well in complete medium (RPMI1640 supplemented with 10% FBS and GlutaMax). Cells were treated in triplicate with bortezomib (dose range 0–10 nm, Janssen Cilag, New Brunswick, NJ, USA) in complete medium at the time of cell seeding and subsequently maintained in culture for an additional 72 h. Cell viability was then assessed over 60 min at 37 °C using the alamarBlue fluorescent dye assay as stated above. Data are expressed as % viability relative to no drug.

### 2.11 Statistical analyses

Survival analyses based on DSG2 subgroups (high versus low on MM PC in BM) were performed using the Kaplan–Meier method with log‐rank tests to assess differences in survival between groups. Multivariable Cox proportional hazards regression models were constructed to estimate the risk of progressive MM/death (progression‐free survival) or death (overall survival) based on DSG2 expression level and/or therapy administered and/or genetic subgroup and/or MS+ versus MS− groupings at diagnosis. Univariable linear regression and Pearson’s correlation analyses were undertaken to test for linear relationships between DSG2 expression levels on MM PC and other blood parameters in MM patients. stata version 14 (StataCorp, College Station, TX, USA) was used for the aforementioned analyses while other statistical analyses (contingency analysis using Fisher’s exact test, Spearman’s correlation analysis and one‐way ANOVA with multiple comparisons and Mann–Whitney test) were performed in prism software (GraphPad, San Diego, CA, USA) v5.04. Test statistics resulting in a *P*‐value < 0.05 were deemed significant.

## 3 Results

### 3.1 DSG2 is expressed by MM PC at the gene and protein level in a distinct subset of MM patients

**Fig. 1:**
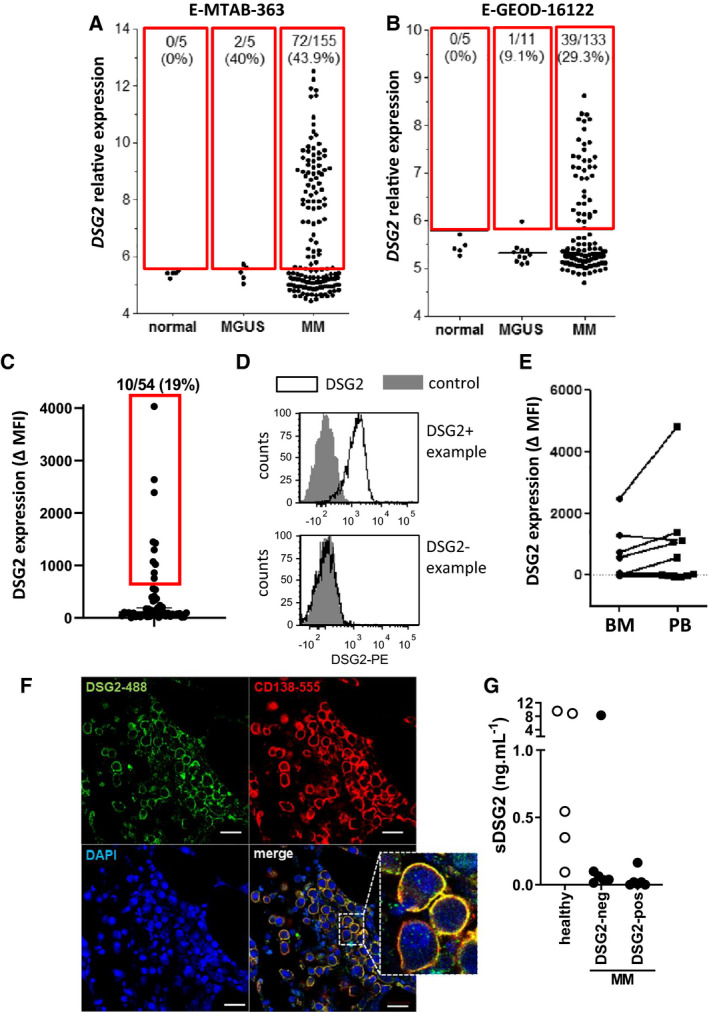
DSG2 is expressed by MM PC at the gene and protein level in a distinct subset of MM patients. (A, B) *In silico* analysis of publicly available microarray datasets E‐MTAB‐363 (A) and E‐GEOD‐16122 (B) was performed. In these studies, RNA was extracted from CD138^+^ PC isolated from BM of normal donors and patients with MM or MGUS, and gene expression levels determined using the Affymetrix U133Plus2.0 platform. Threshold *DSG2* expression values of 5.80 (A) and 5.62 (B) were established as described in Materials and methods, and the proportion of DSG2^+^ samples above this threshold (as shown by the red boxes) determined for each group. (C–E) BM or blood samples from MM patients were analysed by multicolour flow cytometry, gating on viable CD38++/CD138+/CD45lo/CD19− PC. DSG2 expression was quantified as the difference in median fluorescence intensity (ΔMFI) between the DSG2‐stained sample and FMO control. (C) shows all BM samples analysed from newly diagnosed patients (*n* = 54, cut off MFI for inclusion into DSG2^+^ category = 600, as indicated in the red box). Representative histograms are shown in (D). In (E), DSG2 expression by circulating MM PC was also assessed by FACS. (F) Representative BM trephine biopsy from a MM patient stained by immunofluorescence for DSG2 and CD138 and mounted with DAPI as indicated. Scale bar = 20 μm. (G) Serum concentrations of sDSG2 from healthy donors (*n* = 5) and patients with MM (DSG2‐neg *n* = 6 and DSG2‐pos *n* = 7) measured using ELISA.

To assess the expression of *DSG2* in MM PC, we analysed data from two publicly available DNA microarray datasets: E‐GEOD‐16122 [19] and E‐MTAB‐363 [20] that measured gene expression within CD138^+^ BM PC from newly diagnosed MM patients and compared these with PC from normal BM or MGUS patients. Figure 1A,B shows that *DSG2* was expressed by MM PC in a distinct subset of MM patients. Threshold values for DSG2 expression were established for each dataset based on mean + 2SD of the normal controls, and the proportion of DSG2^+^ samples above this threshold was determined. For both datasets, 0/5 normal BM PC samples were classified as DSG2^+^. In contrast, 72/155 of MM patient samples (46.5%) were DSG2^+^ in the E‐MTAB‐363 dataset and 39/133 (29.3%) samples were DSG2^+^ in the E‐GEOD‐16122 dataset. Marginal expression of *DSG2* was noted in 2/5 (40%) MGUS patients in E‐MTAB‐363 and 1/11 (9.1%) MGUS patients in E‐GEOD‐16122. A contingency analysis performed on the pooled data revealed a statistically significant difference in the proportion of individuals with DSG2^+^ PC between normal donors and MM patients (Fisher’s exact test; *P* < 0.05). Interestingly, no other members of the desmosomal cadherin gene family (*DSG1, DSG3, DSC1, DSC2* or *DSC3*) were overexpressed in MM PC in either study (data not shown).

To assess whether DSG2 is also expressed as a surface protein by MM PC, patient BM mononuclear cells were analysed for DSG2 expression by multicolour flow cytometry. MM PC were gated according to a CD38^++^CD138^+^CD45^lo^CD19^‐^ phenotype and DSG2 expression was quantified as the difference in median fluorescence intensity (ΔMFI) between the DSG2‐stained sample and fluorescence minus one (FMO) control. Figure 1C shows that DSG2 cell surface protein was expressed by a proportion of the patients, with 10 of the 54 BM samples analysed being DSG2^+^ (19%) and readily detectable by flow cytometry. Figure 1D shows representative plots of BM samples from patients that are DSG2^+^ or DSG2^‐^.

For 11 patients from whom we had archived material, we were able to examine and directly compare DSG2 expression on stored peripheral blood (PB) circulating CD38^++^CD138^+^CD45^lo^CD19^‐^ (MM PC) against stored BM mononuclear cell MM PC. Figure 1E shows that patients whose MM PC in the BM were DSG2^+^, their MM PC in the PB were also DSG2^+^. In contrast, the majority of patients whose MM PC in the BM lacked DSG2 were similarly DSG2^‐^ in the PB. Interestingly, however, one patient had MM PC in the PB which were DSG2^+^, while their BM counterparts were DSG2^‐^. Immunofluorescence microscopy of a BM trephine biopsy further confirmed co‐expression of DSG2 and CD138 by MM PC for a patient who also tested positive for DSG2 by flow cytometry (Fig. 1F).

An extracellular fragment of DSG2 can be shed from the cell surface via MMP9 [25] and ADAM17 [26] and has been documented to be elevated in the serum of patients with pancreatic cancer [27]. To examine whether soluble DSG2 (sDSG2) is detectable in patients with MM, an ELISA was used to test the serum of 13 MM patients previously identified in Fig. 1C to be either negative or positive for DSG2 on their MM PCs as well as serum from healthy donors. Figure 1G shows that sDSG2 is detectable in a proportion of the donors (range 0–9.5 ng·mL^−1^, comparable to levels identified by Kosanam *et al*. [27]). However, no discernible increase in sDSG2 was identified for the DSG2^+^ MM patients.

### 3.2 DSG2 is expressed by a distinct subset of human MM cell lines

**Fig. 2:**
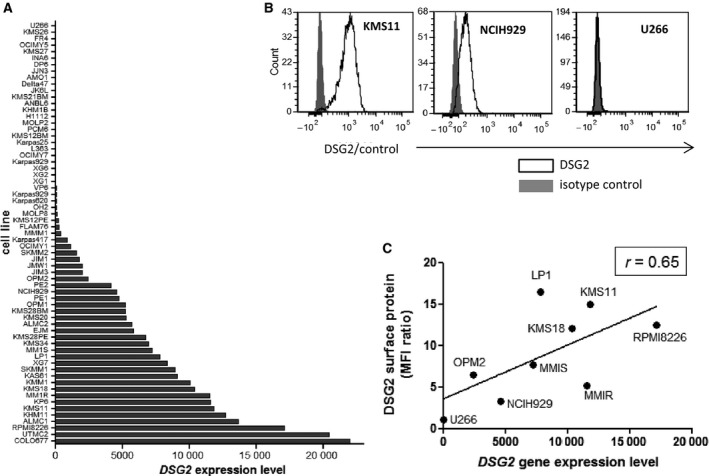
DSG2 expression in a subset of MM cell lines. (A) DSG2 gene expression values for 65 human MM cell lines were extracted from a publicly available RNAseq dataset as described in Materials and methods. Cell lines were ranked according to level of *DSG2* gene expression for simplicity of visualization. (B, C) For nine of the cell lines shown in A, surface expression of DSG2 protein was assessed by flow cytometry. Examples of negative, low and high expression are shown in (B), while the relationship between gene and surface protein for all cell lines analysed is shown in C (Spearman’s correlation coefficient *r* = 0.65).

To extend our analyses of *ex vivo* patient samples, we investigated DSG2 expression in patient‐derived MM cell lines. Initially, gene expression was assessed in a panel of 65 human MM cell lines by interrogating publicly available RNA sequencing data [21] (Fig. 2A). Similar to the patient samples, more than half (55.4%) of the human MM cell lines tested (using an expression threshold of 100) also expressed *DSG2*. For nine of these cell lines, we also measured expression of DSG2 surface protein by flow cytometry (Fig. 2B,C). DSG2 surface protein was readily detectable on cells which expressed *DSG2* mRNA (e.g. KMS‐11, RPMI8226 and NCI‐H929) but was undetectable in the U266 line which had gene expression below the expression threshold (Fig. 2B). Figure 2C suggests a positive correlation between levels of gene expression and levels of surface protein.

### 3.3 DSG2 expression is an independent predictor of poor survival despite association with NSD2 expression

**Fig. 3:**
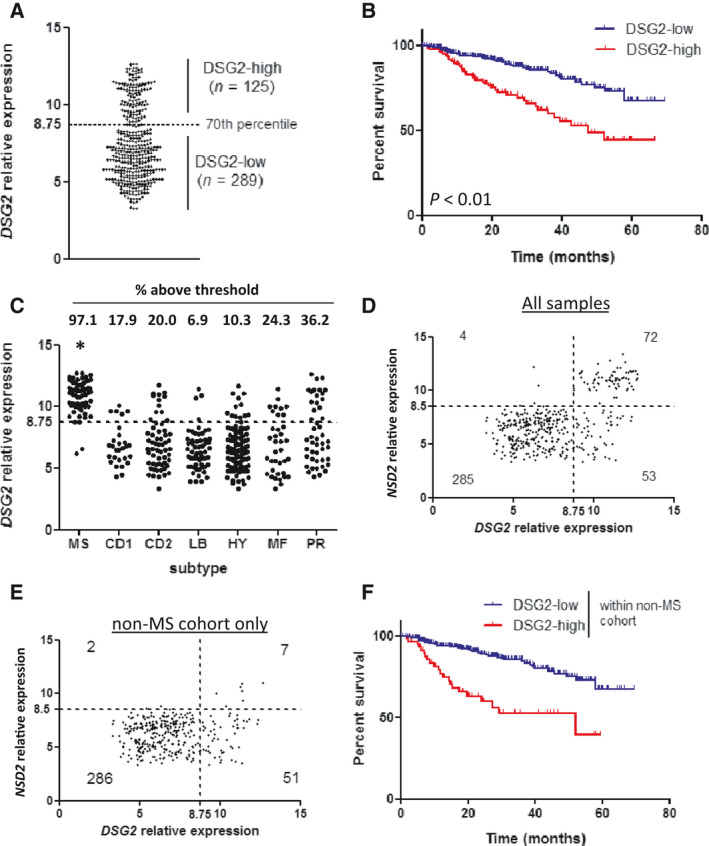
DSG2 expression in MM is strongly associated with reduced survival, independent of NSD2. (A) Microarray dataset GSE4581 was analysed for expression of *DSG2* using probe set 1553105. Visual inspection of the data spread revealed a cluster of samples with elevated *DSG2* expression. A 70/30 percentile split was applied to the data, which cleanly separated these DSG2‐low and DSG2‐high populations, as shown, for further analysis. (B) Overall survival was compared between the DSG2‐low (lower 70%, *n* = 289) and DSG2‐high (upper 30%, *n* = 125) subsets using Kaplan–Meier analysis. *P* < 0.01 (C) Expression of *DSG2* was compared between patients grouped into disease subtypes according to gene expression signatures. *DSG2* expression was significantly greater in the MS subset compared to all others (Kruskal–Wallis test). (D, E) Scatterplots comparing expression of *DSG2* and *NSD2* genes in all samples (D) or non‐MS samples only (E). Dotted lines indicate thresholds for expression based on 70^th^ percentile (*DSG2*) or 80^th^ percentile (*NSD2*). Values represent the number of samples in each quadrant. (F) The non‐MS patient cohort was stratified into *DSG2*‐low and *DSG2*‐high subsets and overall patient survival compared using Kaplan–Meier analysis.

To assess a potential link between DSG2 expression and overall survival of MM patients, we analysed the publicly available gene expression dataset GSE4581, in which CD138^+^ MM PC were purified from the BM of newly diagnosed MM patients using magnetic sorting, and gene expression was subsequently analysed using cDNA microarray [22]. Figure 3A shows that *DSG2* gene expression in this dataset revealed a clear separation of samples into *DSG2*‐high (*n* = 125) and *DSG2*‐low (*n* = 289) groups, with a division based on the 70^th^ percentile of *DSG2* expression. Of note, when these groups were compared by Kaplan–Meier survival analysis, a markedly inferior overall survival (OS) was observed for patients with high *DSG2* expression compared to those with low *DSG2* expression (*P* < 0.001, Fig. 3B). The median OS in the *DSG2*‐high group was 47 months, while the median OS was not reached in the *DSG2*‐low group. Notably, the risk of death was 2.7 times higher in the *DSG2*‐high group (HR 2.69, 95% CI 1.73–4.18, *P* < 0.001; Model 1 in Table S1).

The t(4;14)(q13;q32) translocation is a relatively common genetic event in MM (~ 15%), resulting in overexpression of the histone methyltransferase NSD2 (also known as MMSET or WHSC1), resulting from fusion between *NSD2* and the *IGH* locus [3]. NSD2 overexpression, in turn, deregulates the expression of multiple genes, one of which is *DSG2* [22, 28]. In fact, NSD2 has been shown to directly drive *DSG2* expression in MM cells [29]. As the t(4;14) translocation is an established genetic marker of intermediate to poor prognosis [4], as is the related expression of NSD2 [22], we hypothesized that there may be a link between *DSG2* expression and reduced survival due to its association with NSD2 expression. To address this possibility, we performed further analysis of the GSE4581 dataset. In this dataset, patient samples have been allocated to one of seven subgroups based on a prediction analysis for microarray (PAM) signature by the original study authors; that is MMSET (MS), CCND1 (CD1), CCND3 (CD2), low bone disease (LB), hyperdiploid (HY), MAF/MAFB (MF) and proliferative (PR) [22]. As shown in Fig. 3C, *DSG2* expression was significantly higher in the MS subgroup compared to each of the other subgroups (Kruskal‐Wallis test; *P* < 0.05). Moreover, patients in the MS subgroup were almost uniformly *DSG2*‐high (66/68 patients; 97.1%), using the same threshold for expression as used for the full cohort analysis. Because the dominant feature of the MS subgroup is strong expression of *NSD2* [22], this finding is consistent with the known association between *DSG2* and *NSD2* expression [29]. Importantly though, each of the other six subgroups also harboured a subset of *DSG2*‐high samples, ranging from 6.9% to 36.2% of the patients (Fig. 3C), and *DSG2* retained overall prognostic significance after adjusting for all MM genetic subgroups concurrently (HR 3.03, 95% CI 1.75–5.25, *P* < 0.001; Model 2 in Table S1). Even in patients with hyperdiploidy (HY), which occurs in up to 50% of MM and is associated with a more favourable prognosis [3], high *DSG2* expression in MM PC identifies a subgroup with notably poorer survival (HR 3.21, 95% CI 1.04–9.92, *P* = 0.04). Moreover, high *DSG2* expression identifies a poor prognosis subset of patients in 2 of 4 favourable prognosis genetic subgroups and in the MF (poor prognosis) subgroup, characterized by MAF rearrangements. After statistical adjustments for multiple comparisons, borderline poor prognostic significance of *DSG2* was evident for the MF subgroup and the LB (low bone disease) subgroup. The effect of *DSG2* expression on patient survival in each genetic subgroup before and after adjustments for multiple comparisons is shown in Table S2. These data are the first to suggest that *DSG2* may be a strong predictor of poorer patient survival, independent of cytogenetic risk group.

The detection of *DSG2* in non‐MS subsets suggests that expression of this gene may arise through alternate mechanisms that are independent of *NSD2*. To investigate this, we plotted expression values for *DSG2* against those for *NSD2*. When all samples were included in the analysis, a clear subset co‐expressed both genes at high levels (upper right quadrant in Fig. 3D). On this basis, a threshold for significant expression of *NSD2* was set at 8.5. Unsurprisingly, when this same threshold was applied specifically to the non‐MS samples (Fig. 3E), the vast majority (337/346; 97.4%) fell below the threshold for *NSD2* expression. More importantly, this was also true specifically within the *DSG2‐*high subset, where 51/58 (87.9%) of *DSG2*‐high samples lacked significant co‐expression of *NSD2*. In a Kaplan–Meier analysis of the non‐MS cohort stratified into *DSG2‐*high and *DSG2*‐low groupings, the *DSG2*‐high group again had significantly poorer survival, with an almost four‐fold greater risk of dying compared to those who were categorized as *DSG2*‐low (HR 3.68, 95% CI 2.18–6.22, *P* < 0.001, Fig. 3F and Model 3 in Table S1). These data suggest that *NSD2* is not the only factor that drives *DSG2* expression in MM PC. While the therapy patients received (total therapy 2 or 3) made no difference to overall survival, the predictive ability of *DSG2* was even greater after concurrently adjusting for both MS subset and therapy administered (HR 4.30, 95% CI 2.47–7.48, *P* < 0.001; Model 4 in Table S1).

Next, we analysed RNAseq gene expression data from patients recruited to the coMMpass study through the Multiple Myeloma Research Foundation (MRFF). To determine whether patients with high *DSG2* MM PC expression (based on the 75^th^ percentile) should be initially treated with a particular drug class, we selected 357 patients who were administered either the proteasome inhibitors bortezomib or carfilzomib, or the immunomodulatory agent lenalidomide in frontline regimens but not more than one of these agents in the same regimen. Multivariable Cox modelling demonstrated that overall, high *DSG2* expression within MM PC at diagnosis retained its link with poor prognosis for both progression‐free survival (PFS) (HR 1.38, CI 1.06–1.79, *P* = 0.02) and OS (HR 1.52, CI 1.08–2.14, *P* = 0.02). Interestingly, high *DSG2*‐expressing patients had inferior PFS if treated with either proteasome inhibitor and inferior OS if treated with bortezomib, compared to low *DSG2*‐expressing patients, although the carfilzomib data suffered from high patient dropout. Surprisingly, lenalidomide appeared to abrogate the link of poor prognostic effect with high *DSG2* MM PC expression at diagnosis (PFS: HR 1.20, CI 0.67–2.13, *P* = 0.55; OS: HR 1.00, CI 0.41–2.38, *P* = 0.99) and could suggest a particular benefit in using this or other immunomodulatory drugs for treating *DSG2*‐high patients (Tables S3 and S4).

Finally, using our BM samples from the 54 newly diagnosed MM patients, we observed that high DSG2 expression on the surface of MM PC at diagnosis (determined via flow cytometric analysis, Fig. 1C) again conferred inferior PFS (HR 2.71, 95% CI 0.88–8.37, *P* = 0.08). Surface expression of DSG2 did not linearly correlate with other routinely measured blood parameters, including paraprotein, light chains, β_2_‐microglobulin, haemoglobin, calcium and renal function (Table S5). Moreover, DSG2 expression did not correlate with the plasma cell burden in the BM. Together, these findings clearly suggest that *DSG2* is predictive of the progression‐free and overall survival of newly diagnosed MM patients, independent of *NSD2* expression (and thus, by extension, the t(4;14) translocation), and potentially independent of routinely measured biomarkers of MM activity and/or prognosis. Furthermore, lenalidomide appears to abrogate the poor prognosis of high MM PC *DSG2* expression while bortezomib and possibly carfilzomib worsen prognosis, though these findings should be formally examined in the setting of controlled trials.

### 3.4 Analysis of genes differentially expressed between DSG2^+^ and DSG2^‐^ MM PC

**Fig. 4:**
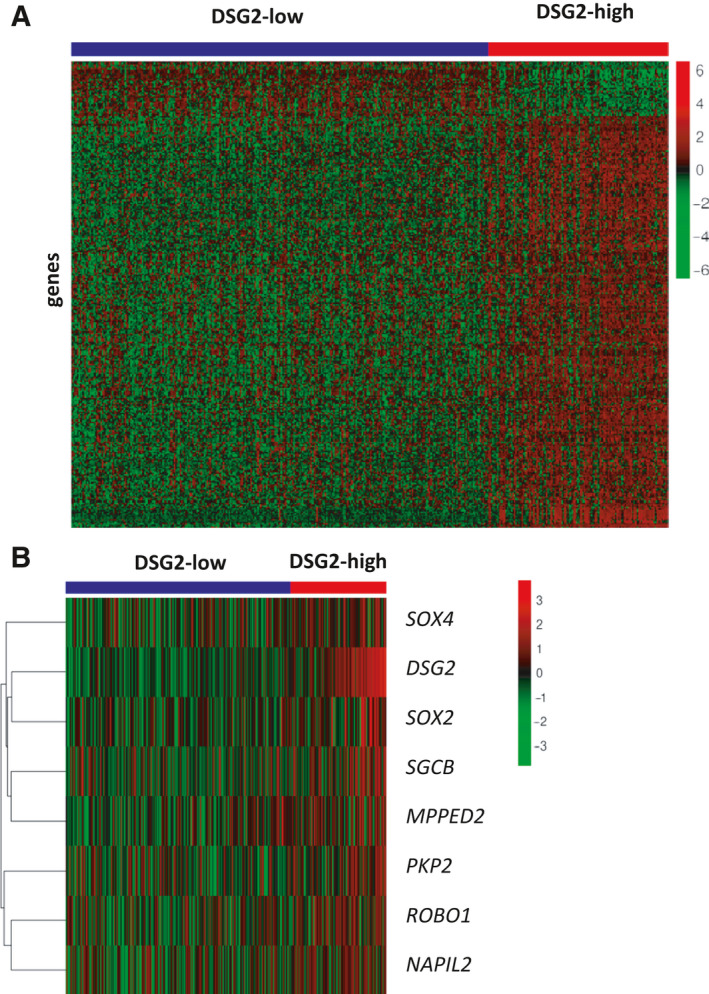
Differential gene expression analysis comparing DSG2‐low and DSG2‐high subsets. Dataset GSE4581 was stratified into DSG2‐low (blue bar) and DSG2‐high (red bar) patient subsets as per Fig. 3, and genes differentially expressed between the two groups were identified and displayed in heatmaps. Clustering of genes displayed in the heatmap was unsupervised and shown as analyses of the entire patient cohort (A), or only the subgroup of patients lacking MMSET expression (MS‐neg; B).

We next compared global gene expression profiles in patient samples defined in Fig. 3 as *DSG2*‐high or *DSG2‐*low. When analysing the entire patient cohort, the *DSG2*‐high and *DSG2*‐low subsets revealed highly divergent gene expression profiles, with 316 significantly differentially expressed genes (Fig. 4A). However, these distinct transcriptional profiles may be driven largely by the t(4;14) translocation and subsequent expression of the NSD2 methyltransferase which is known to regulate hundreds of genes [28]. We therefore also analysed the non‐MS samples separately which revealed seven genes (*SOX4, SOX2, SGCB, MPPED2, PKP2, ROBO1* and *NAPIL2*) differentially expressed between the *DSG2*‐high and *DSG2*‐low subsets (Fig. 4B). Thus, *DSG2* expression in MM appears to arise by two distinct means, either as part of a wider genetic programme induced by NSD2 or as an isolated event induced by unknown mechanisms and not associated with consistent co‐regulation of a large set of other genes.

In light of previous publications demonstrating a correlation between microvascular density and MM progression [30, 31], we compared levels of *DSG2* against the pro‐angiogenic factor *VEGFA*. Interestingly, *VEGFA* levels did not differ between the *DSG2*‐high and *DSG2*‐low patients in the non‐MS cohort (data not shown). However, with *DSG2*, *SOX4* and *SOX2* all previously implicated in tumour angiogenesis [12, 14, 32, 33] factors other than VEGFA may be contributing to the microvascular density in MM, and while we had insufficient patient trephine biopsies to compare DSG2 expression with microvascular density, this will be investigated in future studies.

### 3.5 No detectable role for DSG2 in regulating the growth, survival or major signalling pathways of the KMS‐11 MM cell line

**Fig. 5:**
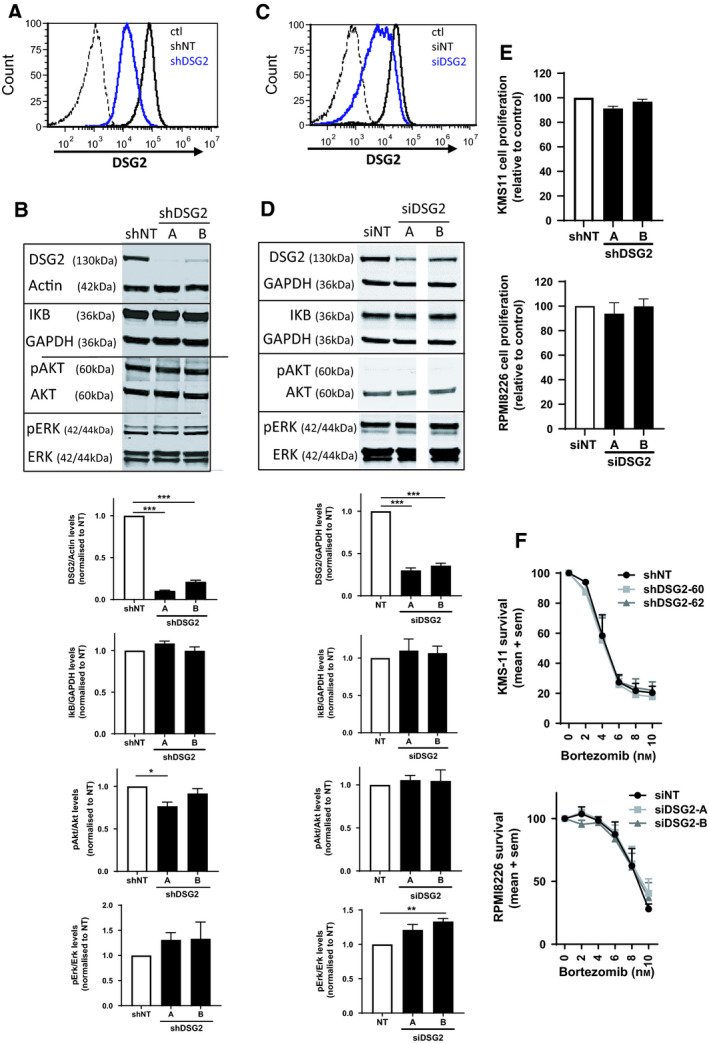
DSG2 knockdown does not affect the major signalling pathways or survival of human MM cells. (A) Flow cytometric analysis of KMS‐11 cells stably expressing nontargeting shRNA (NT, thick black line), DSG2‐targeting shRNA (shDSG2, thick blue line) or isotype control stained cells (ctl, dotted line). (B) Western blot analysis of key signalling proteins in KMS‐11 cells (± shDSG2). Representative blots are shown on the top, while band densities pooled from 3 experiments are shown below; mean ± SEM, one‐way ANOVA with multiple comparisons ***P < 0.001 compared to shNT control. (C) Flow cytometric analysis of RPMI8226 cells transiently expressing nontargeting siRNA (NT, thick black line), DSG2‐targeting siRNA (shDSG2, thick blue line) or isotype control stained cells (ctl, dotted line). (D) Western blot analysis of key signalling proteins in RPMI8226 cells (± siDSG2). Representative blots are shown on the top, while band densities pooled from 3 experiments are shown below, mean ± SEM, one‐way ANOVA with multiple comparisons ***P < 0.001 compared to shNT control. (E) Cell proliferation (metabolic rate) was determined by alamarBlue for KMS‐11 cells (± shDSG2) and RPMI8226 cells (± siDSG2) under normal culture conditions for 72 h. Data are pooled from 3 experiments, mean ± SEM, one‐way ANOVA. (F) KMS‐11 cells (± shDSG2) and RPMI8226 cells (± siDSG2) were treated with bortezomib (0–10 nm) for 72 h prior to cell viability being determined by alamarBlue. Data are normalized to no drug treatment and pooled from 3 experiments, mean ± SEM, two‐way ANOVA.

To begin exploring potential biological functions for DSG2 in MM PC, we used both stable and transient knockdown approaches in two different DSG2^+^ human MM cell lines. First, we validated the effectiveness of short hairpin RNA (shRNA)‐mediated stable knockdown of DSG2 protein by flow cytometric analysis (Fig. 5A) and western blot (Fig. 5B) in the DSG2^+^ KMS‐11 cell line using two different *DSG2‐*targeting shRNA constructs (A and B). When directly compared for activation of major growth and survival signalling pathways, no significant differences were noted in the expression of IκB or the phosphorylation of ERK (Fig. 5B). While a marginal reduction in phosphorylation of AKT was noted with one *DSG2*‐targeting shRNA‐A, this was not observed with the second short hairpin construct (Fig. 5B).

To confirm these results in a second human MM cell line and to validate that the stable knockdown of DSG2 had not caused some compensatory effects to overcome changes in cell signalling, we repeated these experiments in the DSG2^+^ RPMI8226 cells using a transient approach via small interfering RNA (siRNA). The RPMI8226 cell line was deliberately chosen as it represents the non‐MS group by not harbouring the t(4;14) translocation and therefore lacking expression of the NSD2 methyltransferase. Figure 5C,D illustrate the significant reduction of DSG2 on the cell surface as well as total protein following 72 h of siRNA administration using flow cytometry and western blot. These RPMI8226 cells showed similar results to the KMS‐11 cells, with loss of DSG2 conveying little/no effect on protein levels of p‐Akt, total Akt, p‐ERK, total ERK, IKB or GAPDH (Fig. 5D).

We next compared the proliferative rates of the KMS‐11 cells (± shDSG2) as well as RPMI8226 cells (± siDSG2) over 72 h in normal culture conditions. As shown in Fig. 5E, loss of DSG2 does not influence MM cell numbers. Finally, given that the proteasome inhibitor bortezomib is a frontline therapy for myeloma, we examined whether loss of DSG2 could render the cells more susceptible to killing by this drug. Figure 5F shows that bortezomib was not more effective in cancer cell killing following stable knockdown of DSG2 in the KMS‐11 cells or transient knockdown of DSG2 in the RPMI8226 cells.

### 3.6 DSG2 is expressed by endothelial cells within the BM and mediates adhesion with MM PC

**Fig. 6:**
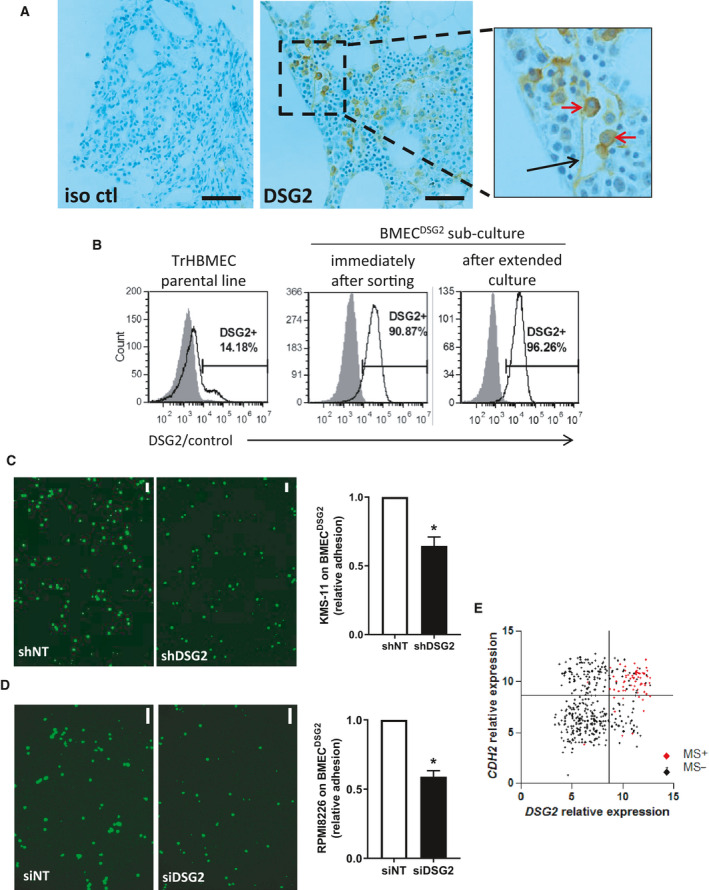
DSG2 promotes the adhesion of MM plasma cells to BM endothelial cells and is co‐regulated with N‐cadherin. (A) BM trephine biopsies from three MM patients were stained for DSG2 by immunohistochemistry; a representative example shows the isotype control (iso ctl) and DSG2‐stained section with a DSG2‐expressing blood vessel (black arrow) and MM PC (red arrows). (B) Expression of DSG2 by the TrHBMEC cell line was assessed by flow cytometry in the parent culture (left); after sorting on DSG2 expression to enrich for BMEC^DSG2^ cells (centre); or after extended passage of the BMEC^DSG2^ cells (right). (C) Adhesion of KMS‐11 cells (± shDSG2) to a monolayer of BMEC^DSG2^ cells for 15 min followed by extensive washing and cell adhesion quantified by imaging the GFP reporter in the KMS‐11 cells. Shown in (C, left image) are representative fluorescent images (scale bar = 100 μm) while (C, right image) shows a summary graph of four independent experiments (Mann–Whitney test *P < 0.05 compared to shNT control). (D) Adhesion of RPMI8226 cells (± siDSG2) to a monolayer of BMEC^DSG2^ cells as above. Representative fluorescent images (scale bar = 100 μm) are shown in (D, left image) while (D, right image) shows a summary graph of three independent experiments (Mann–Whitney test *P < 0.05 compared to siNT control). (E) Gene expression values for *DSG2* and *CDH2* (N‐cadherin) were extracted from dataset GSE4581. Samples in the MS subgroup are shown in red while others (MS‐negative) are shown in black. Quadrants were set visually to highlight the four distinct subsets defined by individual or co‐expression of *DSG2* and *CDH2*.

While reviewing the BM trephine biopsies stained for DSG2 (Fig. 1F), we observed that expression of DSG2 was not limited to the MM PC but was also detectable on blood vessel structures in all three of the BM specimens examined. An example of a DSG2‐expressing blood vessel is shown in Fig. 6A (and for a second MM patient in Fig. S1A). In contrast, in a representative healthy donor BM trephine biopsy we were unable to find a CD31^+^ blood vessel that also stained for DSG2 (Fig. S1B), but could identify DSG2^+^ cells that we predict were haematopoetic progenitor cells. A heterogeneous expression of DSG2 by endothelial cells in the BM is consistent with reports, by us and others, of detectable DSG2 in some, but not all, of the vasculature in normal and cancerous tissues of humans and mice [12, 13, 34, 35]. Further support for heterogeneous expression of DSG2 by BM endothelial cells was obtained by flow cytometric analysis of an immortalized endothelial cell line derived from human BM (TrHBMEC) [15] wherein we identified a distinct subpopulation of DSG2^+^ cells (Fig. 6B; left panel).

Given the well‐established role for DSG2 in cell–cell adhesion via homotypic interactions [12, 14], we went on to test whether DSG2 may be used by the MM PC to bind to BM endothelial cells. To achieve this, first, we FACS sorted the aforementioned DSG2^+^ subpopulation of TrHBMEC cells in Fig. 6B and enriched for endothelial cells that uniformly expressed DSG2 (Fig. 6B; centre and right panels); hereafter referred to as BMEC^DSG2^ cells. Next, we established a confluent monolayer of BMEC^DSG2^ cells and assessed their ability to bind the GFP‐tagged KMS‐11 cells (± shDSG2). Figure 6C shows that stable knockdown of DSG2 significantly attenuated KMS‐11 adhesion to the endothelial cell monolayer when compared to the control cell line. Similar results were observed using the RPMI8226 cell line (± siDSG2) wherein we also observed a ~ 50% reduction in cell adhesion (Fig. 6D). Based on these findings, we propose that a potential biological role for DSG2 on MM PC is to mediate adhesion to BM endothelium.

Of note, we recently demonstrated that the closely related molecule, N‐cadherin (*CDH2*), also mediates adhesion of MM PC to BM endothelial cells [36, 37]. Similar to DSG2, N‐cadherin was found to be expressed on MM PC from a distinct subset of patients. Accordingly, we next examined whether these two cadherins, with apparently similar function, were co‐expressed on MM PC. Figure 6E shows a moderate (*r* = 0.26) but statistically significant (*P* < 0.0001) positive correlation between *DSG2* and *CDH2* gene expression. Notably, however, almost all instances of co‐expression of these two genes occurred within the MS subgroup (highlighted in red in Fig. 6E), with 57/68 (83.8%) of samples from patients in the MS subgroup expressing both *DSG2* and *CDH2*. In contrast, *DSG2* expression was independent of *CDH2* in the majority of non‐MS patients, with 37/346 (10.7%) expressing *DSG2* alone, 97/346 (28.0%) expressing *CDH2* alone and just 22/346 (6.4%) co‐expressing both *DSG2* and *CDH2*. Thus, *DSG2* and *CDH2* may be induced together by the NSD2 methyltransferase in patients with the t(4;14) translocation, but are likely subject to independent regulation in non‐MS subtype MM PC.

## 4 Discussion

In the present study, we identified that DSG2 is a surface protein aberrantly expressed by MM PC in a distinct subset of patients with particularly poor prognosis. The strong association between DSG2 expression and poor prognosis (independent of other routinely measured biomarkers of MM activity, e.g. paraprotein, light chains, β_2_‐microglobulin, haemoglobin, calcium and renal function) suggests an underappreciated functional role for DSG2 in MM pathogenesis. To this end, our functional studies demonstrate that DSG2 mediates adhesive interactions between MM PC and BM endothelial cells. We hypothesize that these interactions may contribute to the dissemination of MM PC, by promoting the extravasation of circulating MM PC from the blood into new sites in the BM.

DSG2 is principally involved in the formation of desmosomal adhesion structures to maintain the integrity of tissues which are subjected to high degrees of mechanical stress, including epithelial tissues and the myocardium (reviewed in [38]). It may therefore seem counterintuitive that DSG2 would be expressed by MM PC, which have not been described to form desmosomes, and do not form a tightly integrated tissue structure requiring the strong adhesive forces that desmosomes provide. However, DSG2 is emerging as a cadherin with many functions additional to those described for desmosome formation. In the context of cancer, DSG2 has been shown to promote the proliferation of colon cancer and non‐small cell lung cancer cells [39, 40], to support vasculogenic mimicry by melanoma cells [14] and protect epithelial cells from apoptosis [9, 10]. These studies in normal and cancerous epithelial cell types raised the possibility that DSG2 may perform similar functions in MM PC. However, our results suggest no measurable effect on proliferation, survival or activation of the NFκB, ERK or AKT signalling pathways in KMS‐11 and RPMI8226 cells. Nor did we observe an increase in soluble DSG2 in the serum of DSG2‐high MM patients, a feature further supported by our observation of the human MM cell lines (e.g. KMS‐11, RPMI8226 and LP‐1) exhibiting only a full‐length version of DSG2 by western blot (data not shown). On the other hand, we have previously shown that DSG2 contributes to homotypic cell–cell adhesion between melanoma cells [14] and endothelial cells [12], both of which lack classical desmosomal structures. Others have also suggested that DSG2 can function as a solitary adhesion molecule outside of desmosomes [8, 11]. With additional information that KMS‐11 and RPMI8226 cells express little to no DSG1, DSG3 or DSC2/3 (data not shown), which would traditionally support desmosome formation, we therefore hypothesized that DSG2 may mediate adhesion to the vascular endothelium via homotypic DSG2‐DSG2 interactions between MM PCs and BM endothelial cells. This possibility was further strengthened by our observation that blood vessels within patient BM biopsies expressed DSG2 on their inner lumen and that the endothelial cell line TrHBMEC, derived from normal human BM, contains a DSG2^+^ population. Strikingly, reducing expression of DSG2 in the MM PCs resulted in a significant decrease in adhesion of KMS‐11 and RPMI8226 cells to a monolayer of DSG2^+^ TrHBMECs (i.e. BMEC^DSG2^). Our observation that adhesion was not completely blocked in these assays was expected as several other adhesion molecules support this process, including integrin α4β1 [41], CD44 [41] and N‐cadherin [36]. On the basis of these functional studies, we propose that a key function for DSG2 on MM PC is to mediate adhesion to DSG2‐expressing endothelial cells. Curiously, DSG2 is not readily detectable on all endothelial cells, with the vasculature of normal and cancerous tissues of humans and mice displaying a heterogeneous profile of this cadherin [12, 13, 34, 35]. With evidence for DSG2 to (a) support the self‐renewal of pluripotent stem cells [42], (b) be co‐expressed with haematopoetic stem/progenitor markers (e.g. CD133 and CD34) [12] and (c) be elevated on endothelial progenitor cells [12], it is tempting to speculate that DSG2 is expressed by vasculature that is either newly formed or experiencing heightened levels of oxidative stress [14]. These observations are supported by the clinical manifestation linked to DSG2 ‘loss of function’ in microvascular endothelial cells identified in patients with systemic sclerosis [13, 43] and of microvascular density being associated with MM progression [30, 31]. While we did not observe a difference in *VEGFA* levels between the *DSG2*‐high and *DSG2*‐low patients in the non‐MS cohort, other genes associated with tumour vascularization (e.g. *SOX4* and *SOX2* [32, 33]) were co‐expressed with *DSG2*.

While we are yet to determine the precise contribution of DSG2 to the pathology of MM, it is our contention that DSG2 assists in the coordinated responses of cell‐to‐cell communication via cell–cell adhesion (as proposed above), but this can also occur via cytokine release and/or extracellular vesicle interactions. Exosomes (small extracellular vesicles, 30–150 nm, loaded with various cargo including DNA, RNA, lipids and proteins [44]) serve as intercellular messengers with documented roles in pathological processes, including MM [45]. Wang *et al*. [45] demonstrated that MM‐derived exosomes prime the BM microenvironment for enhanced angiogenesis and immunosuppression via the activation of JNK and STAT3 in the BM ECs. Interestingly, a role for DSG2 in exosomes and cancer progression has been identified in squamous cell carcinoma with DSG2 promoting the secretion of exosomes that contain pro‐mitogenic cargo such as IL‐6 [46, 47], a known contributor to myeloma development and progression [48]. A comprehensive analysis of DSG2 levels, microvascular density and exosomes in MM patients is beyond the resources of this study but will be required to definitively answer these questions.

One of the defining features of MM is the presence of multiple lesions at sites throughout the skeleton at the time of diagnosis [16], suggesting that MM PC dissemination is an intrinsic feature of this cancer. Notably, elevated numbers of circulating tumour cells are a predictor of disease progression from MGUS and smouldering MM [49, 50, 51] and disease relapse following therapy [52, 53, 54], independent of tumour burden, suggesting the importance of haematogenous spread in MM disease progression. The process of dissemination of MM PC is thought to be similar to that of metastasis in solid tumours, requiring adhesion to vascular endothelial cells to enable transendothelial migration and facilitate spread to secondary sites via the peripheral circulation [55, 56]. To this end, integrin α_4_β_1_, CD44 and N‐cadherin have been shown to play a role in the adhesion of primary MM PC and MM cell lines to endothelial cells *in vitro* [36, 41]. Moreover, blockade or loss of N‐cadherin or CD44 is sufficient to inhibit the homing of MM PC from the peripheral blood to the BM in mouse models of MM [36, 57], highlighting the importance of MM PC adhesion to endothelial cells in the dissemination process. Our present observations suggest that circulating MM PC may also use DSG2 to bind to vascular endothelium to exit the bloodstream and seed new sites. Further studies are required to determine whether blocking DSG2 function may be a novel approach to reduce MM disease progression.

Since there are now multiple adhesion molecules identified that promote the adhesion of MM PC to endothelial cells, the question arises: what is the relationship between them? To explore this further, we focussed on N‐cadherin, which our group has previously studied in detail in the context of MM [36, 37]. In our previous studies, we demonstrated that N‐cadherin is expressed by MM PC in a distinct subset of patients; that expression is associated with reduced progression‐free and overall survival; and that N‐cadherin knockdown reduces the adhesion of MM PC to BM endothelial cells and limits disease progression in an animal model. Here, we explored the relationship between expression of the genes for N‐cadherin and DSG2 and observed that some patients’ MM PC expressed only *DSG2* while others only expressed *CDH2*. This supports the concept that distinct adhesion mechanisms evolve independently in individual patients. However, we also noted that these genes could be co‐expressed, and this occurred almost exclusively in patients within the MS subgroup. This is in keeping with previous studies demonstrating that expression of both genes can be regulated by NSD2 in human t(4;14)‐positive MM cell lines [29, 58], strongly suggesting that NSD2 is responsible for the overexpression of DSG2 and N‐cadherin in these cell lines. This coordinated induction of multiple adhesion molecules, each with distinct binding partners and biological functions, may collectively contribute to the more aggressive, disseminated and treatment‐resistant disease phenotype that is characteristic of t(4;14) myeloma [5, 6, 59]. This finding also raises the intriguing possibility that DSG2 and N‐cadherin may physically interact as heterodimers on the MM PC surface in t(4;14)‐positive MM patients. In this regard, another classical cadherin, E‐cadherin, has recently been shown, using atomic force microscopy, to form *cis* dimers with DSG2, thus demonstrating the capacity of heterodimer formation between desmosomal cadherins and classical cadherins [60]. Determining whether N‐cadherin and DSG2 undergo similar interactions, and how these impact on cellular adhesion to the endothelium, awaits further study.

While our data support the hypothesis that *DSG2* is regulated by NSD2 in t(14;14)‐positive patients, the factors that induce overexpression of DSG2 in MM PC which lack the t(4;14) translocation remain to be identified. Of relevance to this, several lines of evidence suggest that DSG2 expression may be induced as part of a stem cell‐associated genetic programme. For example, within the haematopoietic compartment, we previously demonstrated that DSG2 is almost ubiquitously expressed by CD34^+^CD90^+^CD117^+^CD38^‐^ stem cells in normal human BM, with expression being progressively down‐regulated in more differentiated haematopoietic cell subsets [12]. Specifically, within the B‐cell lineage, DSG2 expression remained detectable on a subset of pro‐B cells but was lost in the pre‐BI, pre‐BII, immature B‐cell and mature B‐cell subsets. As DSG2 has also been shown to be expressed by other stem/progenitor cell populations [12, 61], it is our contention that the expression of DSG2 in myeloma lacking the t(4;14) translocation reflects a partial de‐differentiation of plasma cells to a haematopoietic stem cell‐like phenotype. Our comparison of gene expression patterns within the non‐MS patient subset identified seven genes differentially expressed between *DSG2*‐high and *DSG2*‐low patient subsets, and two of these are transcription factors that have been associated with either pluripotent stem cells (*SOX2*) [62] or haematopoietic stem/progenitor cells (*SOX4*) [63, 64]. Further studies are required to determine whether expression of these transcription factors is responsible for the induction of DSG2 expression in t(4;14)‐negative MM patients.

The results presented here reveal that DSG2 may be a clinically useful prognostic biomarker in MM. Being a surface protein detectable by flow cytometry, DSG2 could be readily assessed as part of routine diagnostic analysis of BM specimens to provide valuable prognostic information at the time of diagnosis. The ability to recognize high‐risk MM at diagnosis is becoming increasingly important as personalized treatment approaches gain momentum, seen, for example, with the use of upfront tandem autologous stem cell transplantation for genetic high‐risk MM resulting in improved clinical outcomes [65]. Furthermore, so‐called response‐adapted approaches are being examined in clinical trials, where therapy is altered based on objective measures such as BM minimal residual disease during treatment [66]. Advances in optimizing MM treatment require novel biomarkers to inform decision‐making, and it is likely that no single biomarker will be sufficient to effectively guide therapeutic decisions in all patients. Our findings suggest that DSG2 could independently add to the prognostic utility of established genetic risk factors, and our future work focuses on identifying the drugs that are most effective against DSG2‐expressing MM PCs.

## 5 Conclusions

In conclusion, our studies suggest that DSG2 may be a molecule of great relevance in MM biology. DSG2 plays a nonredundant role in the adhesion of MM PC to endothelial cells and is thus a potential therapeutic target for reducing or preventing disease dissemination and progression. In addition, the clear link between DSG2 expression and poor prognosis implicates this surface protein, which is a readily measurable and clinically useful prognostic biomarker. Future work will focus on confirming the importance of DSG2 to BM homing and disease progression *in vivo* and on developing DSG2‐based assays to more accurately stratify MM patients according to disease risk at the time of diagnosis and thereby improve clinical outcome.

## Conflicts of interest

The authors declare no conflict of interest.

## Author contributions

LME, KV, ACWZ, CTW‐B and CSB conceived and designed the project; LME, KV, MJZ, MD, LYT, KKMM, BMW, BWE, CTW‐B and CSB acquired the data; LME, KV, SMP, CTW‐B and CSB analysed and/or interpreted the data; LME, KV, CTW‐B and CSB wrote the paper. All authors read and approved the final manuscript.

### Peer Review

The peer review history for this article is available at https://publons.com/publon/10.1002/1878‐0261.13055.

## Supporting information


**Fig. S1**. DSG2 expression on MM PCs and BM ECs. BM trephine biopsies from a MM patient in (A) and a healthy donor in (B), stained for DSG2 by immunohistochemistry; a representative example shows the isotype control (iso ctl) and DSG2‐stained section with a DSG2‐expressing blood vessel (black arrow) and MM PC (red arrows). In (B), a serial section was stained for CD31 to identify BM vasculature (black arrow) and DSG2 positive progenitor cells (green arrow).

**Table S1**. Summary of the multivariable Cox regression models based on dataset GSE4581 [Zhan et al, Blood 2006] for the effect of DSG2 expression on overall survival.
**Table S2**. The effect DSG2 expression on patient overall survival in each genetic subgroup using dataset GSE4581 and univariable Cox regression modelling.
**Table S3**. Multivariable Cox regression model of the coMMpass study data for progression‐free survival by DSG2 expression level and frontline therapy.
**Table S4**. Multivariable Cox regression model of the coMMpass study data for overall survival by DSG2 expression level and frontline therapy.
**Table S5**. Correlation of DSG2 expression on patient MM PC against routinely measured blood parameters.

## Data Availability

The data that support the findings of this study are openly available at https://doi.org/10.1002/gcc.20668 ref #19, https://doi.org/10.1093/bioinformatics/btt124 ref #20, https://doi.org/10.1182/blood.V122.21.1914.1914 ref #21 and https://doi.org/10.1182/blood‐2005‐11‐013458 ref #22.
