# Supplementary material for: Desmoglein‐2 expression is an independent predictor of poor prognosis patients with multiple myeloma
Source: Mol Oncol. 2021 Jul 24;16(6):1221–40. doi: 10.1002/1878-0261.13055 (PMC8936512; doi:10.1002/1878-0261.13055)
Supplement: Supplementary file 1 — Fig. S1. DSG2 expression on MM PCs and BM ECs. BM trephine biopsies from a MM patient in (A) and a healthy donor in (B), stained for DSG2 by immunohistochemistry; a representative example shows the isotype control (iso ctl) and DSG2‐stained section with a DSG2‐expressing blood vessel (black arrow) and MM PC (red arrows). In (B), a serial section was stained for CD31 to identify BM vasculature (black arrow) and DSG2 positive progenitor cells (green arrow). [file MOL2-16-1221-s002.pdf]

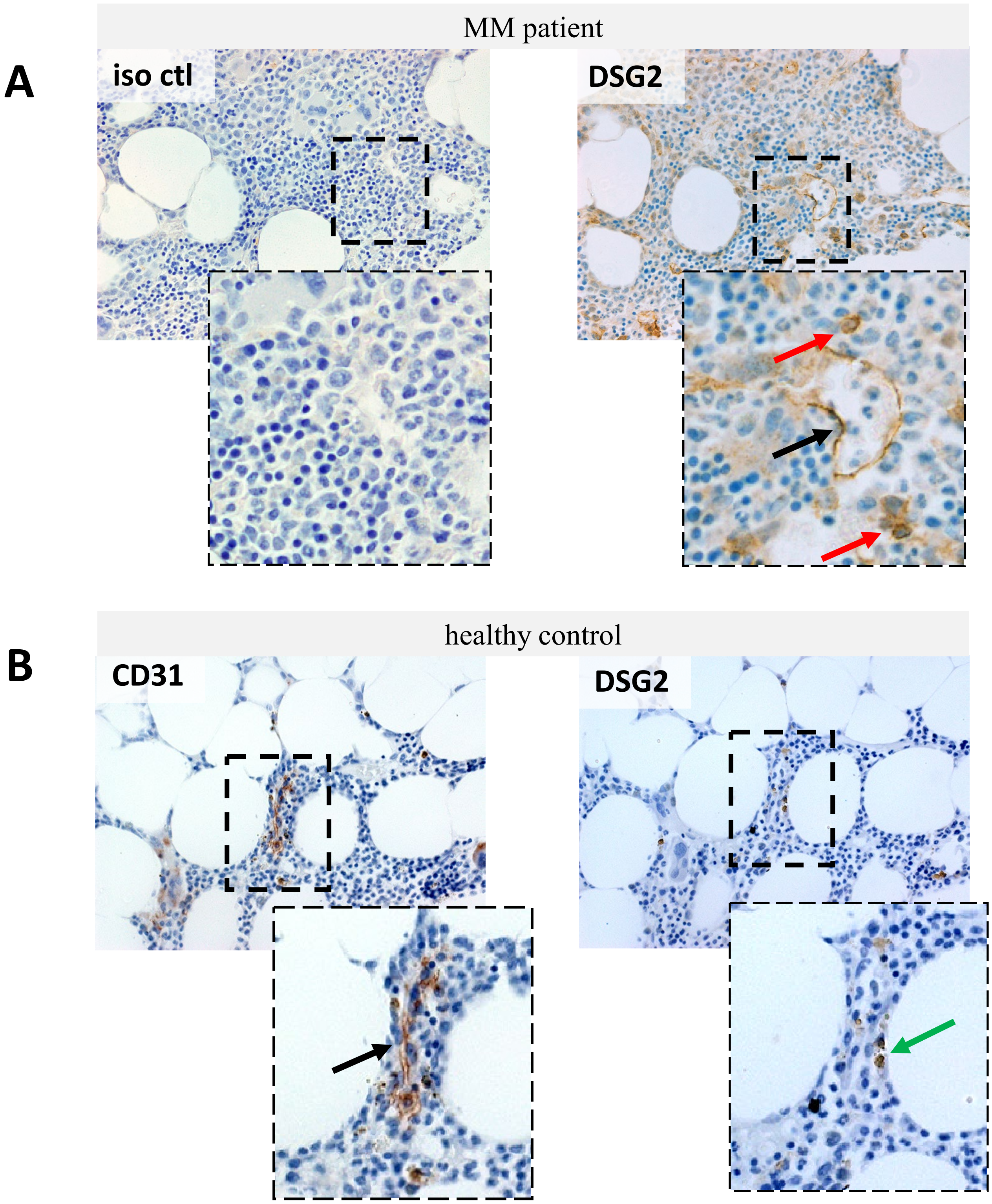

Supplementary Figure S1: DSG2 expression on MM PCs and BM ECs. BM trephine biopsies from a MM patient in (A) and a healthy donor in (B), stained for DSG2 by immunohistochemistry; a representative example shows the isotype control (iso ctl) and DSG2 stained section with a DSG2-expressing blood vessel (black arrow) and MM PC (red arrows). In (B), a serial section was stained for CD31 to identify BM vasculature (black arrow) and DSG2 positive progenitor cells (green arrow).
