# Supplementary material for: Desmoglein‐2 expression is an independent predictor of poor prognosis patients with multiple myeloma
Source: Mol Oncol. 2021 Jul 24;16(6):1221–40. doi: 10.1002/1878-0261.13055 (PMC8936512; doi:10.1002/1878-0261.13055)
Supplement: Supplementary file 2 — Table S1. Summary of the multivariable Cox regression models based on dataset GSE4581 [Zhan et al, Blood 2006] for the effect of DSG2 expression on overall survival. Table S2. The effect DSG2 expression on patient overall survival in each genetic subgroup using dataset GSE4581 and univariable Cox regression modelling. Table S3. Multivariable Cox regression model of the coMMpass study data for progression‐free survival by DSG2 expression level and frontline therapy. Table S4. Multivariable Cox regression model of the coMMpass study data for overall survival by DSG2 expression level and frontline therapy. Table S5. Correlation of DSG2 expression on patient MM PC against routinely measured blood parameters. [file MOL2-16-1221-s001.pdf]

Supplementary Table S1. Summary of the multivariable Cox regression models based on dataset GSE4581 [Zhan et al, Blood 2006] for the effect of DSG2 expression on overall survival

Cox Model	Model Covariates	Hazard Ratio	95% Confidence Interval	p value
Model 1 (all patients)	• DSG2 expression level	2.69	1.73 – 4.18	< 0.001
Model 2 (all patients)	• DSG2 expression level<br>• Genetic subgroup	3.03	1.75 – 5.25	< 0.001
Model 3 (non-MS cohort)	• DSG2 expression level<br>• MS cohort	3.68	2.18 – 6.22	< 0.001
Model 4 (all patients)	• DSG2 expression level<br>• MS cohort<br>• Therapy	4.30	2.47 – 7.48	< 0.001

Supplementary Table S2. The effect DSG2 expression on patient overall survival in each genetic subgroup using dataset GSE4581 and univariable Cox regression modelling

	Genetic Grouping	Prognostic Significance	DSG2 (high/low)	Hazard Ratio	95% Confidence Interval	p value	Adjusted Confidence Interval (99%)	Adjusted p value
CD-1 (n=28)	Cyclin D1 and cyclin D3: t(11;14) and t(6;14)	Low-risk	5 / 23	6.60	0.91 – 47.56	0.06	0.49 – 88.46	0.37
CD-2 (n=59)	Cyclin D1 and cyclin D3: t(11;14) and t(6;14)	Low-risk	12 / 48	0.62	0.07 – 5.18	0.66	0.04 – 10.07	4.00
HY (n=116)	Hyperdiploidy	Low-risk	12 / 104	3.21	1.04 – 9.92	0.04	0.73 – 14.14	0.26
LB (n=58)	Low bone disease	Low-risk	4 / 54	9.08	1.65 – 50.13	0.01	0.96 – 85.75	0.07
MF (n=37)	MAF: t(14;16) and t(14;20)	High-risk	9 / 28	8.76	1.70 – 45.20	0.01	1.01 – 75.70	0.06
MS (n=68)	MMSET: t(4;14)	High-risk	66 / 2	Indeterminate	Indeterminate	Indeterminate		
PR (n=47)	Proliferation related	High-risk	17 / 30	2.00	0.79 – 5.11	0.15	0.59 – 6.85	0.87

Supplementary Table S3. Multivariable Cox regression model of the coMMpass study data for progression-free survival by DSG2 expression level and frontline therapy

**Therapy**	**Median PFS (months)**		**Multivariable Cox Regression** *(DSG2-high vs DSG2-low)*
	DSG2-low	*DSG2-high*	
Bortezomib	22	15	HR 1.49, CI 1.10 – 2.03, P = 0.01
Carfilzomib	NR	26	HR 2.41, CI 0.64 – 9.10, P = 0.19
Lenalidomide	21	22	HR 1.20, CI 0.67 – 2.13, P = 0.55

Supplementary Table S4. Multivariable Cox regression model of the coMMpass study data for overall survival by DSG2 expression level and frontline therapy

**Therapy**	**Median OS (months)**		**Multivariable Cox Regression** *(DSG2-high vs DSG2-low)*
	DSG2-low	*DSG2-high*	
Bortezomib	58	36	HR 1.86, CI 1.27 – 2.71, P < 0.01
Carfilzomib	NR	NR	HR 1.17, CI 0.20 – 7.04, P = 0.86
Lenalidomide	60	61	HR 1.00, CI 0.41 – 2.38, P = 0.99

Supplementary Table S5. Correlation of DSG2 expression on patient MM PC against routinely measured blood parameters.

Biomarker	Linear Regression (p value)	Pearson Correlation (r value, p value)
Paraprotein	0.151	- 0.091, 0.543
κ light chain	0.549	- 0.090, 0.549
λ light chain	0.488	0.104, 0.488
κ:λ ratio	0.655	-0.067, 0.655
Haemoglobin	0.131	- 0.224, 0.131
White cell count	0.518	0.097, 0.518
Platelet count	0.700	0.058, 0.700
β_2-microglobulin	0.425	-0.027, 0.860
Urea	0.738	0.051, 0.738
Creatinine	0.558	0.088, 0.558
Calcium	0.929	-0.013, 0.929
Albumin	0.143	-0.217, 0.143
Lactate dehydrogenase	0.168	-0.135, 0.365
Plasma cell percentage (marrow aspirate)	0.154	-0.055, 0.716
Plasma cell percentage ($CD138^+$ IHC of trephine)	0.442	-0.145, 0.331
